# Supplementary material for: Genome-wide diversity and global migration patterns in dromedaries follow ancient caravan routes
Source: Commun Biol. 2020 Jul 16;3:387. doi: 10.1038/s42003-020-1098-7 (PMC7366924; doi:10.1038/s42003-020-1098-7)
Supplement: Supplementary file 1 — Supplementary Information [file 42003_2020_1098_MOESM1_ESM.pdf]

Supplementary Materials

Supplementary Figures

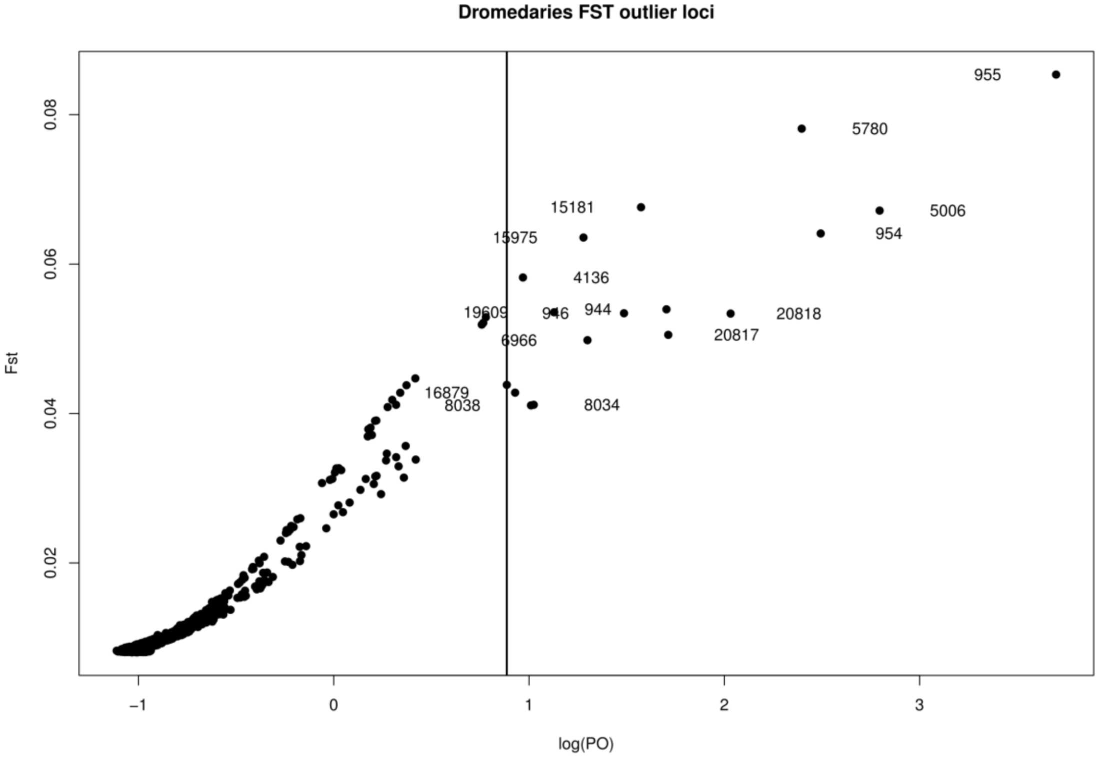

Supplementary Figure 1. F_{ST} outlyers. Sixteen loci putatively under selection detected using Bayescan (q-value lower than 5%). PO = posterior odds [28].

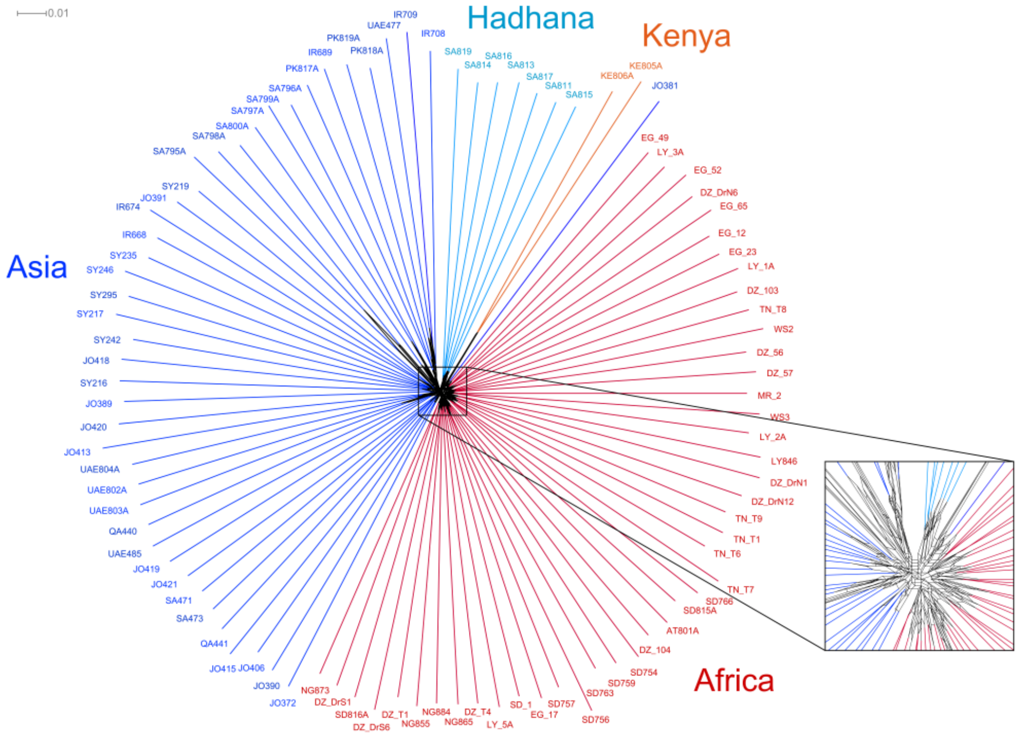

Supplementary Figure 2. SplitsTree network. Phylogenetic network calculated with SplitsTree using Neighbour-net, with a zoom to visualize the split between Africa, Asia, *Hadhana* and Kenyan dromedaries.

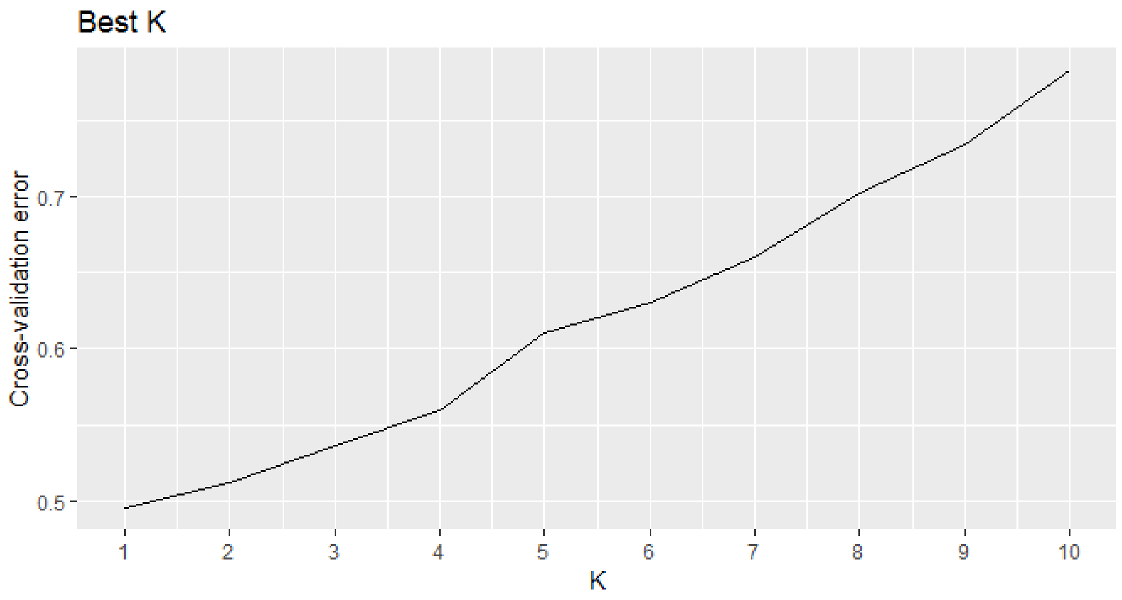

Supplementary Figure 3. Admixture´s best K according to cross-validation error from K=1 to K=10.

Supplementary Tables

Supplementary Table 1. Allelic richness (AR), observed and expected heterozygozities (H_O, H_E) and inbreeding coefficients (F_{IS}) for the total population, per continent and per c ountry. AR calculations for Africa and Asia were corrected for 46 individuals minimum; A R calculations for countries were corrected for one individual minimum. SD corresponds to the standard deviation.

N	Countries	AR	SD	H_O	SD	H_E	SD	F_{IS}	SD
95	All	-	-	0.25	0.17	0.27	0.17	0.06	0.17
46	Africa	1.97	0.16	0.25	0.18	0.26	0.17	0.058	0.2
49	Asia	1.98	0.13	0.25	0.17	0.27	0.17	0.063	0.2
1	MR	1.26	0.44	0.26	0.44	NA	NA	NA	NA
2	WS	1.26	0.28	0.25	0.33	0.26	0.3	-0.06	0.56
11	DZ	1.26	0.19	0.25	0.21	0.26	0.19	0.03	0.3
5	TN	1.26	0.21	0.25	0.25	0.26	0.21	0.01	0.38
4	NG	1.26	0.22	0.25	0.27	0.26	0.23	-0.01	0.42
9	SD	1.25	0.2	0.23	0.21	0.26	0.2	0.07	0.34
2	KE	1.21	0.28	0.19	0.3	0.23	0.31	0.02	0.59
5	LY	1.26	0.21	0.26	0.25	0.27	0.22	0.01	0.4
6	EG	1.26	0.21	0.25	0.24	0.26	0.21	0.02	0.37
1	AT	1.26	0.44	0.26	0.44	NA	NA	NA	NA
7	SY	1.26	0.2	0.23	0.22	0.26	0.2	0.08	0.37
12	JO	1.26	0.19	0.24	0.2	0.27	0.19	0.07	0.3
15	SA	1.26	0.18	0.25	0.2	0.26	0.18	0.03	0.26
2	QA	1.27	0.29	0.27	0.34	0.27	0.3	-0.1	0.56
5	UAE	1.26	0.21	0.25	0.25	0.26	0.21	0.02	0.39
5	IR	1.27	0.21	0.25	0.24	0.27	0.21	0.05	0.4
3	PK	1.26	0.25	0.26	0.3	0.26	0.25	-0.04	0.48

NA due to low number of individuals

Supplementary Table 2. AMOVA. Groups correspond to Africa and Asia, populations correspond to countries.

**Source of variation**	**d.f.**	**Sum of squares**	**Variance components**	**Percentage**	***P* value**
Among groups	1	3404.275	9.45527	0.45	< 0.005
Among populations within groups	15	35787.158	19.85303	0.94	< 0.005
Among individuals within populations	78	169914.609	91.32516	4.32	< 0.05
Within individuals	95	189595.5	1995.74211	94.30	< 0.005

Fixation Indices	
F_{IS}	0.04376
F_{SC}	0.00942
F_{CT}	0.00447
F_{IT}	0.05700

Supplementary Table 3. Pairwise *F_{ST}* values between dromedary populations from different countries including a minimum of three individuals (below the diagonal) and their significance levels (+) $P < 0.05$ (above the diagonal).

	DZ	TN	NG	SD	LY	EG	SY	JO	SA	UAE	IR	PK
DZ		-	-	-	-	+	+	+	+	+	+	+
TN	0.0045		-	+	-	-	+	+	+	-	-	-
NG	0.0008	0.0102		-	-	-	+	+	-	-	-	-
SD	0.0083	0.0130	0.0037		-	-	-	-	-	-	-	-
LY	0.0002	0.0021	0.0044	0.0082		-	+	-	-	-	-	-
EG	0.0051	0.0061	0.0085	0.0110	0.0015		+	-	+	+	+	-
SY	0.0121	0.0128	0.0122	0.0148	0.0060	0.0097		-	-	+	-	-
JO	0.0060	0.0070	0.0066	0.0111	0.0019	0.0052	0.0039		-	-	-	+
SA	0.0090	0.0101	0.0101	0.0141	0.0080	0.0106	0.0087	0.0050		-	-	-
UAE	0.0148	0.0158	0.0152	0.0184	0.0136	0.0160	0.0142	0.0047	0.0131		-	-
IR	0.0210	0.0223	0.0207	0.0240	0.0157	0.0201	0.0062	0.0074	0.0156	0.0070		-
PK	0.0290	0.0328	0.0311	0.0343	0.0230	0.0267	0.0217	0.0160	0.0251	0.0137	0.0030	
