# Supplementary material for: Genome-wide diversity and global migration patterns in dromedaries follow ancient caravan routes
Source: Commun Biol. 2020 Jul 16;3:387. doi: 10.1038/s42003-020-1098-7 (PMC7366924; doi:10.1038/s42003-020-1098-7)
Supplement: Supplementary file 2 — Description of Additional Supplementary Files [file 42003_2020_1098_MOESM2_ESM.pdf]

Description of additional supplementary files

Supplementary Data 1. Detailed sample information.

Supplementary Data 2. Number of paired-end ddRAD reads mapping unambiguously to either dromedary or Bactrian camel genome for all 123 dromedary and Bactrian camel samples.

Supplementary Data 3. Identification of genes located within 200kbp upstream and downstream of loci under putative selection, with respective functions of loci under putative selection. In bold are depicted the genes related to immunity.
